# Expression of Claudins in Preneoplastic Conditions of the Gastrointestinal Tract: A Review

**DOI:** 10.3390/cancers15164095

**Published:** 2023-08-14

**Authors:** Abderrahman Ouban, Tarek Ziad Arabi

**Affiliations:** 1 Department of Pathology, College of Medicine, Alfaisal University, Riyadh 11533, Saudi Arabia; 2 College of Medicine, Alfaisal University, Riyadh 11533, Saudi Arabia; tarabi@alfaisal.edu

**Keywords:** preneoplastic conditions, Barrett’s esophagus, metaplasia, cirrhosis, adenoma, inflammatory bowel disease, claudins, tight junctions, oncogenesis

## Abstract

**Simple Summary:**

Recent evidence has emerged highlighting the role of claudins in the development of cancers of gastrointestinal organs. Accordingly, studies have further analyzed the role and expression of claudin in preneoplastic gastrointestinal conditions, including Barrett’s esophagus, sessile serrated polyps, inflammatory bowel disease, and others. Several claudins, mainly -1, -2, -3, -4, and -18, have been identified as aberrantly expressed in these conditions. Furthermore, studies have highlighted their prognostic role in patients. Identifying how claudin expression changes in preneoplastic conditions may allow for early diagnosis and treatment. Additionally, it may unlock the door for potential therapeutic molecular targets.

**Abstract:**

Premalignant lesions of the gastrointestinal tract are a group of disorders which act as the harbinger of malignant tumors. They are the ground-zero of neoplastic transformation, and their identification and management offer patients the best opportunity of blocking the progress of cancer. However, diagnoses of some of these conditions are hard to make, and their clinical importance is difficult to assess. Recent reports indicated that several claudin proteins have altered expressions in many cancers, including esophageal, gastric, colon, liver, and pancreatic cancers. The early identification of the aberrant expression of these proteins could lead to the early diagnosis and management of gastrointestinal tumors. Specifically, claudins -1, -2, -3, -4, and -18 are frequently overexpressed in gastrointestinal preneoplastic lesions. These altered expressions have shown clinical value in several tumors, providing diagnostic and prognostic information. In this article, we review the literature on the aberrant expression of claudins in preneoplastic lesions of the gastrointestinal tract. Additionally, we summarize their diagnostic and prognostic implications.

## 1. Introduction

The barrier function of cells is operated by the epithelia, which form a protective layer between the body’s interior milieu and the outside environment [1,2]. At the heart of this barrier function are the tight junction strands, composed of the occludin, zona-occluden, desmosomes, and claudins’ proteins. Tight junctions regulate the permeability of the epithelial barrier and limit passage through paracellular space [3].

In addition to their barrier role in protecting the underlying structures, tight junctions help regulate cellular proliferation and differentiation [4,5,6,7]. Tight junctions also ensure the maintenance of cellular polarity, a critical element in maintaining proper cellular communication and signaling [8,9,10]. Recent mounting evidence points to signaling pathways which converge on tight junction positions and interact with the proteins forming those tight junctions [10,11]. The aberrant expression of these tight junction proteins may play critical roles in establishing alternative signaling and the possible activation of neoplastic pathways which converge upon the cell, resulting in its transformation [12]. Furthermore, several studies have shown that claudins in cancer exhibit changing expression levels [4,13,14,15,16], with evidence presented from the manipulation of claudin levels in in vitro and animal studies [4,13,14], resulting in decreased cell motility, invasion, and metastases. Collectively, the above evidence of claudins’ postulated roles in cancer development and progression suggests the great potential of these proteins in the fight against cancer and its complications.

Early markers of neoplastic transformation may provide the opportunity for early intervention and management. Recent reports have pointed to changing levels of claudins in preneoplastic gastrointestinal disorders. Early markers of gastrointestinal neoplasia are needed because of the difficulty of assessing the extent and degree of the mucosal disease, which is a critical guide in management [17,18], and because both endoscopy and histopathology have failed to provide an accurate estimate of the risk of neoplastic transformation in some gastrointestinal preneoplastic conditions [18,19]. While genomic assays have been assessed as foretellers of gastrointestinal cancers [20,21], no comprehensive reviews are currently available analyzing the proteomic counterparts. This study is a step in that direction.

## 2. Claudin Expression in Esophageal Preneoplastic Conditions

In a study analyzing the expression of claudins in esophageal lesions, Gyorffy and co-workers reported the increased expression of claudin-1 in squamous cell carcinoma compared with normal esophageal mucosa (Table 1). In their study, claudins -3 and -4 were also significantly elevated in Barrett’s esophagus and adenocarcinoma compared to its expression in the foveolar epithelium. Claudin-2, on the other hand, had significantly increased expression in esophageal adenocarcinoma compared with its expression in Barrett’s esophagus [22].

Abu-Farsakh and co-workers showed that claudin-2 is highly expressed in esophageal adenocarcinoma and its premalignant precursors [23]. They showed that there is more significant expression in esophageal adenocarcinoma than in high-grade dysplasia, Barret esophagus, and columnar dysplasia [23] (Table 2). No associations with clinicopathologic attributes of tumors were found [23].

While assessing other claudin proteins’ expression levels in esophageal adenocarcinoma and its precursors, claudin-3 was significantly increased in Barrett’s esophagus compared to normal esophaguses in several studies [24,25,26] (Table 2). On the other hand, strong claudin-4 immunoreactivity was reported in most Barrett’s samples [24]. This included all high- and low-grade dysplasia in Barrett’s. The same study also reported that most esophageal adenocarcinoma cases and its metastases exhibited a similar reaction pattern of claudin-4 to the one seen in Barrett’s esophagus. While both claudins -3 and -4 exhibited similar reaction percentages and patterns in both Barrett’s esophagus and adenocarcinoma cases, claudin-7 showed a different result, where all Barrett’s esophagus and low-grade dysplasia cases were reactive to claudin-7 antibody, and the majority showed strong staining (Table 2) [24,27]. However, high-grade dysplasia, adenocarcinoma, and metastatic cases showed a lower percentage of cases exhibiting strong claudin-7 immunoreactivity (only 50% of cases), with the rest of the cases showing no changing levels [24,27].

**Table 2:** Various gastrointestinal preneoplastic conditions exhibit abnormal claudin expression.

Site	Preneoplastic Lesions	Claudins Expression	Methodology	Notes	References
Esophagus	Barrett’s esophagus	Claudins -1, -3, and -4 ↑	IHC	Esophageal adenocarcinoma has stronger expression than Barrett’s Claudins -3 and -4, via immunohistochemistry and oligonucleotide microarray	[22,24]
		Claudin-2 ↑	IHC	-	[23]
		Claudin-18 ↑↑	PCR and Western blot	-	[28]
	Low-grade dysplasia	Claudin-7 ↑↑	IHC	-	[24]
	High-grade dysplasia, adenocarcinoma, and metastatic tumors	Claudin-7 ↔	IHC	-	
Stomach	Intestinal metaplasia	Claudins -1, -3, -4, -5, and -7 ↑ Claudin-18 ↓	IHC	-	[29,30,31,32]
	Early dysplasia	Claudins -1, -3, and -5 ↑ Claudins -4 and -7 ↑↑ Claudin-18 ↑↑	IHC, IFC, and PCR	Especially claudin-18.2 splice variant, in diffuse gastric carcinoma	[29,30,31,32,33,34]
Colon	SSA/P MVHP APC/Claudin-1 Mice	Claudin-1 ↑	IHC and PCR	-	[35,36,37]
	Adenomatous Polyps	Claudin-1 ↑	IHC and IFC	-	[38,39]
		Claudin-7 ↓	Western blot, PCR, IHC, and IFC	Correlates with *p53* levels Hypermethylation of claudin-7	[40,41,42]
	Inflammatory bowel disease	Claudins -1, -2, -3, and -4 ↑	IHC, IFC, Western blot, and PCR	Expression correlated with inflammatory activity and with activated form of β-Catenin Increased resistance to anoikis through increased Src.	[7,43,44,45,46]
Liver	Cirrhosis	Claudins -1 and -7 ↑	IHC and Western blot	Hepatocellular carcinoma arising in cirrhotic regions has even higher expression Intensity of expression is unrelated to chronic inflammation	[47]
		Claudins -4 and -7 ↑	IHC, Western blot, and IFC	Intensity of expression correlates with grade of fibrosis Intensity of expression is unrelated to chronic inflammation	[48]
Pancreas	Hyperplastic lesions→Hyperplastic foci→Adenomas→Borderline tumors→non-invasive carcinomas→invasive carcinomas	Claudin-1 ↓	IHC and PCR	-	[49,50]
	Hyperplastic lesions→Hyperplastic foci→Adenomas→Borderline tumors→non-invasive carcinomas→invasive carcinomas	Claudin-4 ↑	IHC and PCR	-	
	IPMN	Claudins -2, -4 and -18 ↑	IHC	Highest expression of claudin-2 seen in adenomas in both IPMN and MCN Claudin-2 expression decreased with increased grade Claudin-4 expression increased with increased grade Cancer arising in IPMN and MCN showed strong claudin-18 expression in poorly differentiated regions IPMN and MCN lesions with mild dysplasia exhibited weaker claudin-18 expression Claudin-2 expression decreased with increased histological grade in IPMN and MCN lesions Claudin-4 expression increased with increased histological grade in IPMN and MCN lesions	[51]
	MCN	Claudins -2, -4, and -18 ↑	IHC		

IPMN: intraductal papillary mucinous neoplasms; MCN: mucinous cystic neoplasms; IHC: immunohistochemistry; IFC: immunofluorescence; PCR: polymerase chain reaction; ↑↑: greatly increased; ↑: increased; ↓: decreased.

Claudin-18 was the dominant tight junction protein in Barrett’s esophagus in a study utilizing RT-PCR to assess the expression of 21 claudin proteins in esophageal lesions [28]. This increase was also paralleled by marked protein expression on immunoblots (Table 2). In contrast, in the same study, this expression was diminished in esophageal squamous epithelia from healthy volunteers. Transfecting MDCK II cells with claudin-18 raised its electrical resistance and decreased, selectively, paracellular permeability to sodium and hydrogen, thus contributing to the greater acid resistance of Barrett’s esophagus [28]. On the other hand, in at least one-third of esophageal adenocarcinoma cases, claudin-18 is also over-expressed [52]. 

In summary, claudins -1, -2, -3, -4, -7, and -18 were found to be increased in Barret’s esophagus and low- and high-grade dysplasia (Table 1). Claudins -4 and -18 have the highest increase in Barrett’s, and claudin-7 is most elevated in low-grade dysplasia (Table 2). Claudin-7 expression exhibits no change from normal epithelia in high-grade dysplasia, adenocarcinoma, and metastatic tumors (Table 2). 

## 3. Claudin Expression in Gastric Preneoplastic Conditions

During intestinal metaplasia in gastric mucosa, which is a documented pre-neoplastic condition of the gastric epithelium, claudins’ expression patterns undergo a change where the expression levels of claudins -1, -3, -4, -5, -7, and -18 are increased (Table 2) [29,30,32]. As the metaplastic process turns dysplastic, the above claudin expression levels remain increased, most significantly with claudin -4 and -7 (Table 1 and Table 2). The authors reported increased mRNA of claudins -4 and -7 in a panel of gastric cancer cell lines as well. However, it was noted that the expression levels of claudins -1, -3, -4, -5, and -7 in diffuse gastric carcinoma are decreased [29,53], whereas that of claudin-18 is increased [32,33,34]. 

Claudin-18′s role in gastric carcinogenesis is increasingly under the spotlight. While earlier reports have indicated that mice deficient in claudin-18 experienced the neoplastic transformation of gastric mucosa [31,32,33], more recent reports indicated the overexpression of this protein, specifically the claudin-18 splice variant 2 (claudin-18.2) in gastric adenocarcinomas [32]. While the results may seem contradictory, it was interesting to note that the reported downregulation of claudin-18.2 expression occurred more frequently in the intestinal variant than in the diffuse variant of gastric cancers (significant expression in 46% versus 75%) [32]. Furthermore, the authors have shown that this variant of the protein was also highly expressed in lymph nodes and distant metastatic lesions of gastric adenocarcinoma [32]. This overexpression proved to be of critical clinical significance, where zolbetuximab, an IgG1 monoclonal antibody, could bind to claudin-18.2 and induce gastric cancer cell death via antibody-dependent and complement-dependent cytotoxicity [33]. This highly specific anti-claudin 18.2 monoclonal antibody demonstrated further efficacy in phase II and phase III SPOTLIGHT trials with reported improvements in both progression-free survival (PFS) and overall survival (OS) [34]. 

In summary, claudins -1, -3, -4, -5, and -7 were found to be expressed in both intestinal metaplasia and early dysplasia. Claudins -4 and -7 are more strongly expressed in early dysplastic gastric lesions than in intestinal metaplastic lesions (Table 1). Claudin-18 was upregulated in the diffuse form of gastric adenocarcinoma (Table 1 and Table 2). 

## 4. Claudin Expression in Colonic Preneoplastic Conditions

In 2012, Bezdekova and co-workers reported strong claudin-1 expression in adenomas and adenocarcinomas of the colon [35]. Membranous staining, typical for nontumorous epithelium, switched to combined membranous/cytoplasmic expression in both adenomas and adenocarcinoma in a significant number of cases. Furthermore, this membranous/cytoplasmic expression re-localization was significantly more common in the adenocarcinoma group (87% of cases) compared with the adenoma group (51%). 

Claudin-1 was identified in a gene expression profiling study as the most significant differentiating gene between sessile serrated adenomas/polyps (SSA/P) and micro-vesicular hyperplastic polyps (MVHP). The immunohistochemical analysis of claudin-1 expression in BRAF B600E mutant SSA/P, MVHP and BRAF wild-type MVHP revealed statistically significant correlation between claudin-1 expression and the BRAF V600E for both SSA/PO and MVHP in comparison to wild-type polyps [36]. 

In another study analyzing the role of claudin-1 in adenoma formation in adenomatous polyposis coli (APC) in mice, APC-Cldn1 mice showed significantly increased colonic tumor growth, size, and decreased survival. These mice also demonstrated the inhibition of mucosal defense genes and the sharp upregulation of pro-inflammatory genes, specifically IL-23/IL-17 signaling. This may provide the background for an early onset of adenoma formation in APC-Cldn1 mice [37]. 

Patients with inflammatory bowel disease live with increased risk of neoplastic transformation. Several studies have provided evidence that the upregulation of several claudin proteins contributes to tumorigenesis in ulcerative colitis, including claudins -1, -2, -3, and -4 [43,44,45]. Bhat and co-workers have reported that claudin-1 upregulation in colitis is mediated by tumor necrosis factor (TNF)-α [46]. This may explain the increased resistance to anoikis seen in colorectal cancer cells as a result of the link between TNF-α and claudin-1 [7]. 

Moreover, claudin-7 was found to be deregulated in colonic adenocarcinoma and its precursors. Claudin-7 was found to be significantly downregulated in colon cancer samples [40]. Silencing *Rab25*, a cancer suppressor, reversed the effects of claudin-7 expression, increasing cellular proliferation, invasion, and p-Src and mitogen-activated protein kinase. The above may strongly suggest a role for claudin-7 as a tumor suppressor and that its loss promotes EMT in an *Rab25*-dependent manner [40]. In a second study assessing the role of claudin-7 in colorectal cancer and its precursors, claudin-7 expression was positively correlated with *p53* levels in different stages of adenoma–carcinoma sequence in the colon [54]. The etiology through which claudin-7 may play a role in colorectal tumorigenesis may be related to *p53* binding to the claudin-7 promoter region and regulating its expression. The above may point out the tumor suppressor function of claudin-7 in a *p53*-dependent manner, which may mediate colorectal carcinogenesis induced by *p53* deletion or mutation [54]. Yet, another reason for the tumor-suppressor role played by claudin-7 in the colorectum may be related to the anti-inflammatory role played by this tight junction protein. Severe intestinal inflammation in the small intestine of mice was induced by the controllable deletion of claudin-7 [41]. And finally, in a study of 26 colorectal adenomas compared to 90 invasive CRCs (stage I-IV) in addition to their lymph node metastases, immunohistochemistry revealed decreased claudin-7 expression in 62% of adenomas and in 80% of cases of colorectal cancers, compared to their adjacent non-neoplastic epithelia [42]. The hypermethylation of claudin-7 promoter was detected in 20% of CRCs with low claudin-7 expression [42]. 

In summary, CLDN-1 is expressed in all preneoplastic lesions of colonic adenocarcinoma, including SSA/P, MVHP, APC mutations, adenomatous polyps, and inflammatory bowel disease (Table 1 and Table 2). In inflammatory bowel disease, claudins -1, -2, -3, and -4 were expressed across the spectrum of IBD disease, including active disease, IBD-associated adenoma, and neoplastic transformation emanating from the condition (Table 1 and Table 2). Furthermore, this expression correlated well with the inflammatory activity of the disease. Claudin-7, on the other hand, was reported as decreased in adenomatous polyps.

## 5. Claudins Expression in Hepatic Preneoplastic Condition

In a study assessing the expression of claudins in liver cirrhosis patients, Tsujiwaki and co-workers reported the increased expression of claudin-4 and -7 in cirrhotic livers and correlated this increase in expression with the grade of fibrosis, but not with the inflammatory activity in the surrounding liver tissue of chronic hepatitis [48]. In addition to claudin-4 and -7, claudin-1 also was significantly elevated in cirrhosis compared to non-cirrhotic liver [47]. The authors reported that in samples where hepatocellular carcinoma developed in a background of cirrhosis, even higher claudin-1 levels were present, whereas claudin-7 showed decreased levels in those same samples [47]. Similar to the findings of Tsujiwaki and co-workers, they reported that hepatitis C virus status, with its chronic inflammation, did not impact claudin-1 and -7 levels in cirrhotic livers [47].

In summary, claudins -1, -4, and -7 are positively expressed in cirrhosis of the liver. Hepatocellular carcinoma regions arising from cirrhotic lesions express even stronger expression levels of these proteins.

## 6. Claudins Expression in Pancreatic Preneoplastic Conditions

In 2005, Tsukahara and co-workers reported claudin-1 expression in hyperplastic lesions in the pancreas in 50% (3 out of 6) hyperplastic foci, 82% (14 out of 17) adenomas, 30% (3 out of 10) borderline tumors, 2 out of 6 (33%) non-invasive carcinomas, and 1 out of 5 invasive pancreatic cancers (20%), producing a statistically negative association with progression of malignancy. In contrast, claudin-4 had the reverse trend in the same lesions, producing a positive association with histologically progressive pancreatic tumors [49]. 

In a second study analyzing the expression of claudins in intraductal papillary mucinous neoplasms (IPMNs) and mucinous cystic neoplasms (MCNs), both considered definitive premalignant pancreatic lesions, Lee and co-workers reported that both IPMNs and MCNs were positive for claudins-2, -4, and -18 in similar percentages, albeit there was slightly increased intensities of the expression of all above claudins in IPMN than in MCN [51]. The highest claudin-2 expression was seen in adenomas in both IPMN and MCN lesions. Claudin-2 expression decreased with increased histological grade in both IPMN and MNC lesions, whereas claudin-4 showed the opposite trend [51] (Table 2). The expression of claudin-18 was similar to claudin-4, where one of three IPMN adenomas showed negative expressions of claudin-18, while seven of eight IPMN carcinomas strongly expressed claudin-18. In MCN, 8 of 13 adenomas did not express or had weak expression for claudin-18, whereas all seven carcinomas showed strong claudin-18 expression [51]. 

In their work on pancreatic cell lines and IPMNs, Tsutsumi and co-workers reported similar findings to those of Lee and co-workers [51] and Tsukahara and co-workers [49], where claudin-4 mRNA expression levels were significantly higher in IPMN borderline neoplasms, carcinoma in situ, and invasive pancreatic carcinoma compared with the expression in IPMN adenomas, in a four-way comparison [50]. When the authors divided the tumors between high-grade IPMNs (including borderline, carcinoma in situ, and invasive cancers) and low-grade IPMNs (adenomas), significantly elevated levels of claudin-4 mRNA were seen in the high-grade group when compared to the IPMN adenomas [50]. 

In summary, claudins -2, -4 and -18 were positively expressed in all pancreatic preneoplastic lesions, including hyperplastic foci, adenomas, IPMN, and MSN lesions, with different trends (Table 1 and Table 2). Claudin-1 exhibited a negative association with progression of preneoplastic lesions, whereas claudin-4 showed the opposite association (Table 1 and Table 2). On the other hand, claudins -2, -4, and -18 showed positive association in both IPMN and MCN lesions, albeit with stronger expression in IPMN than in MCN.

## 7. Mechanisms of Aberrant Claudin Expression in Gastrointestinal Preneoplastic Conditions

**Figure 1:**
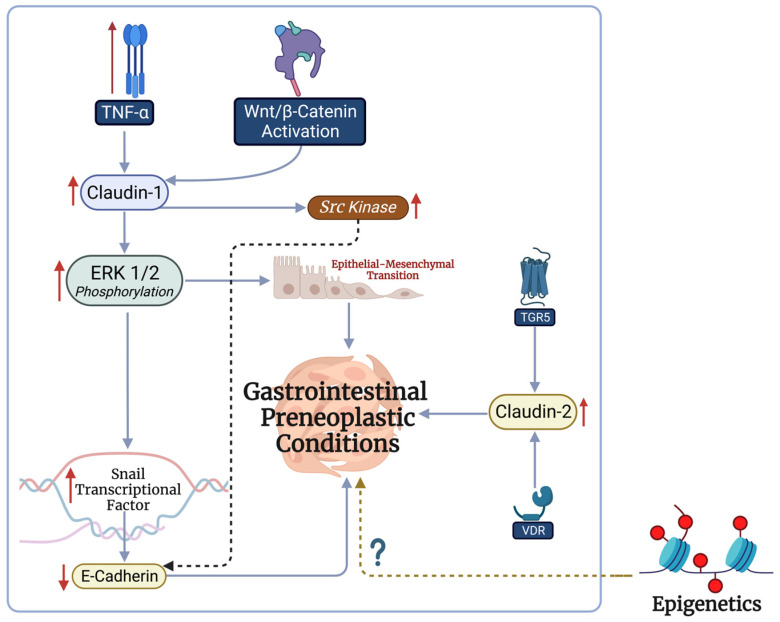
TNF- α, Wnt/β-catenin, bile acid receptors, and, possibly, epigenetics play a key role in the development of gastrointestinal preneoplastic conditions. This figure was generated using biorender.com.

Several mechanisms have been described as mediators underlying the aberrant expression of claudin proteins in gastrointestinal preneoplastic conditions (Figure 1). In this section, we summarize these mechanisms and identify their therapeutic utility.

TNF-α, a prominent pro-inflammatory cytokine, is a key mediator of inflammatory processes in IBD [46]. Furthermore, IBD patients with increased levels of TNF-α are at increased risk of developing colon cancer [43]. TNF-α has been shown to promote claudin-1 expression in colorectal cells [46]. Subsequently, claudin-1 activates the c-Abl-Ras-Raf-1-ERK1/2 pathway, which mediates epithelial–mesenchymal transition and, eventually, cancer [55]. ERK goes on to activate the Snail transcription factor and inhibit E-cadherin expression, a transmembrane protein which connects epithelial cells, in both tumorous and nontumorous cells [6,35,56,57]. Inhibiting Snail using monoclonal antibodies has shown promising outcomes by inhibiting tumor growth and metastasis and promoting tumor-specific tumor-infiltrating lymphocytes in melanoma [58]. Furthermore, claudin-1 modulates Src kinase expression, which promotes cell motility, epithelial–mesenchymal transition, and mesenchymal protein expression [7]. Similarly, suppressing Src inhibits mesenchymal protein expression and increases E-cadherin expression.

The Wnt/β-catenin signaling pathway also plays a key role in the modulation of claudins and neoplastic processes. The pathway has been shown to significantly promote the expression of claudin-1 in colonic cancer cells [59]. Demonstrating its possible therapeutic utility, Hseu et al. found that *Antrodia camphorate* prevents cell migration and epithelial–mesenchymal transition via the suppression of claudin-1 and Wnt/β-catenin [60]. 

In esophageal preneoplastic conditions, bile acids have been shown to promote claudin-2 expression through vitamin D receptors (VDR) and Takeda G-protein-coupled receptor-5 (TGR5) [23]. Claudin-2 expression increases intracellular permeability, promoting cellular changes which could lead to metaplasia and Barrett’s esophagus [23]. Targeting VDR and TGR5 could unlock potential opportunities for the treatment of Barrett’s esophagus.

Emerging evidence has also highlighted the role of epigenetics in abnormal claudin expression in gastrointestinal neoplasms. For example, microRNA-421 promotes the proliferation and metastasis of gastric cancers by inhibiting claudin-1 [61]. This mechanism is likely mediated by the inhibition of apoptosis through reduced caspase-3 expression [62]. DNA hypomethylation has also been associated with claudin-4 overexpression in pancreatic ductal adenocarcinoma [63]. Similar mechanisms could possibly allow for the development of preneoplastic conditions; however, this has not yet been studied, to the best of the authors’ knowledge. Future studies should aim to identify the epigenetic mechanisms involved in gastrointestinal preneoplastic conditions, allowing for the discovery of a wide spectrum of therapeutic targets. Collectively, TNF-α, Wnt/β-catenin, and bile acid receptors play a key role in the development of gastrointestinal preneoplastic conditions and could be targeted in the treatment of such conditions.

## 8. Conclusions

This study provided a review of the unique signature of claudin proteins in preneoplastic lesions of the gastrointestinal tract. The majority of the reported claudins in this study are overexpressed, suggesting possible oncogenic roles for them in preneoplastic lesions (Table 1 and Table 2). Few of the claudins are knocked down in those lesions, including claudin-18 in the stomach, claudin-7 in the colon, and claudin-1 in the pancreas. 

Claudin-7 expression in the colon strongly suggests a tumor-suppressor role for this gene, mediated by *Rab25* [40] or *p53* [54], and accomplished by hypermethylation [42]. On the other hand, claudin-3 and -4 expressions in high-grade gastric dysplasia–early gastric carcinoma predicted higher incidence rates of synchronous or metachronous gastric carcinomas [31].

Claudins have proven valuable in predicting the survival of cancer patients. For example, low claudin-4 expression is significantly associated with improved survival in intermediate-type growth pattern gastric carcinoma [64]. Contrarily, positive claudin-23 expression is predictive of better overall survival in gastric cancer patients [65]. However, besides claudin-1 in the colon and claudins-3, -4, and -18 in the stomach, no other studies have analyzed the prognostic role of claudins in gastrointestinal preneoplastic conditions. Studies analyzing the prognostic implications of claudins in gastrointestinal preneoplastic conditions could guide future treatment and allow for more the individualized treatment of high-risk individuals.

Although most studies have only identified claudins through biopsies, some have demonstrated the possibility of detection using more clinically practical methods. In the colon, Rabinsky and co-workers reported that the overexpression of claudin-1 in premalignant colonic lesions could be detected via endoscopic examination with a peptide marked by near-infrared beams [39]. Furthermore, Hollandsworth et al. demonstrated the utility of claudin-1-directed fluorescence-guided surgery in mouse models of colon cancer [66]. Both preclinical and clinical studies are needed to expand on the methods of claudin detection without the need for biopsies.

From the management perspective, this review of the literature provides several examples in which the expression of claudins in preneoplastic gastrointestinal conditions had therapeutic value. These include the recent discovery of compounds which may modulate the expression of the claudins, such as claudin-1 in the colon [60], and consequently halt the progression of cancer. Another such compound, zinc [67], is currently in a phase 1 pilot clinical trial because it was found to increase claudin-7 expression in Barrett’s esophagus and prevent the development of esophageal adenocarcinoma. The above should provide more impetus to thoroughly assess claudin expression in precancerous conditions.

## Figures and Tables

**Table 1:** The aberrant expression of various claudins in gastrointestinal preneoplastic conditions.

Preneoplastic Lesions	CLDN1	CLDN2	CLDN3	CLDN4	CLDN5	CLDN7	CLDN18
Esophageal	↑	↑	↑	↑/↑↑	-	↔/↑↑	↑↑
Gastric	↑	-	↑	↑/↑↑	↑	↑/↑↑	↑↑
Colonic	↑	↑	↑	↑	-	↓	-
Hepatocellular	↑	-	-	↑	-	↑	-
Pancreatic	↓	↑	-	↑	-	-	↑

CLDN: claudin; ↑↑: greatly increased; ↑: increased; ↔: no changes; ↓: decreased.
